# Advancements in Improving Selectivity of Metal Oxide Semiconductor Gas Sensors Opening New Perspectives for Their Application in Food Industry

**DOI:** 10.3390/s23239548

**Published:** 2023-12-01

**Authors:** Jolanta Wawrzyniak

**Affiliations:** Faculty of Food Science and Nutrition, Poznań University of Life Sciences, 60-624 Poznań, Poland; jolanta.wawrzyniak@up.poznan.pl

**Keywords:** metal oxide semiconductor gas sensors, E-nose, electronic nose, thermally modulated MOS gas sensor, heterostructures, nanostructures, nanomaterials, doping with noble metals, conducting polymers, food safety, pattern recognition algorithms

## Abstract

Volatile compounds not only contribute to the distinct flavors and aromas found in foods and beverages, but can also serve as indicators for spoilage, contamination, or the presence of potentially harmful substances. As the odor of food raw materials and products carries valuable information about their state, gas sensors play a pivotal role in ensuring food safety and quality at various stages of its production and distribution. Among gas detection devices that are widely used in the food industry, metal oxide semiconductor (MOS) gas sensors are of the greatest importance. Ongoing research and development efforts have led to significant improvements in their performance, rendering them immensely useful tools for monitoring and ensuring food product quality; however, aspects related to their limited selectivity still remain a challenge. This review explores various strategies and technologies that have been employed to enhance the selectivity of MOS gas sensors, encompassing the innovative sensor designs, integration of advanced materials, and improvement of measurement methodology and pattern recognize algorithms. The discussed advances in MOS gas sensors, such as reducing cross-sensitivity to interfering gases, improving detection limits, and providing more accurate assessment of volatile organic compounds (VOCs) could lead to further expansion of their applications in a variety of areas, including food processing and storage, ultimately benefiting both industry and consumers.

## 1. Introduction

In recent years, both food safety and quality have been matters of particular concern. According to relevant statistics, in 2020, more than 30 per cent of the world population was affected by moderate to severe food insecurity [https://www.fao.org/state-of-food-security-nutrition/2021/en/ (accessed on 18 September 2023)]. This insecurity can be caused by various factors such as the presence of pesticide residues, prohibited additives, allergens, pathogens, and their toxic metabolites. It not only poses a serious threat to human health, but also imposes certain restrictions on the development of the food industry. As it is necessary to ensure food safety at every stage of the production process, scientists are constantly searching for new analytical methods and equipment aimed at detecting hazards in various food products, as well as to eliminate the threats and conditions conducive to their occurrence. Volatile compounds are responsible not only for the specific flavor and aroma of foods and beverages, but they may also indicate spoilage, contamination, or the presence of harmful substances.

Gas detection is widely used in the food industry to ensure the safety and quality of food products. Metal oxide semiconductor (MOS) gas sensors play an important role in the detection and quantification of specific gases in the surrounding environment. They provide real-time data, which is essential in the food industry, thus allowing companies to ensure that food products meet quality and safety standards. MOS gas sensors are characterized by high sensitivity in detecting volatile components. This is a significant advantage, as they can be used for the detection and monitoring of volatile organic compounds at low concentrations (<ppm levels) [1]. Interest in these sensors is increasing due to their high repeatability, quick response, miniaturization, low cost, and durability [2,3]. However, they also have some limitations, among which the most significant is their low selectivity. This is associated with the fact that upon exposure to a mixture of volatile compounds the output signal of a single sensor carries a limited amount of information, because its response is the sum of signals generated by individual substances and it is impossible to determine which part of the signal corresponds to individual substances [4,5]. This cross-sensitivity results in poor sensor selectivity, especially for ketones and alcohols, to which sensors respond with similar signal intensity [6,7]. As a result, when using a single gas sensor, the quantitative assessment is limited to systems containing only one known type of volatile compound.

In view of the great demand for high-performance gas sensors, researchers are trying to eliminate the disadvantages of these devices. This study provides a comprehensive scientific overview of semiconductor metal oxide gas sensors, referring to selected examples of their applications in the food industry. It discusses principles of their operation, various materials used to their manufacturing, and diverse applications. The aim of the study was to present a detailed overview of the advancements, challenges faced by users and future prospects of MOS gas sensors.

## 2. MOS Gas Sensors—Design and Operation

### 2.1. Design

**Figure 1:**
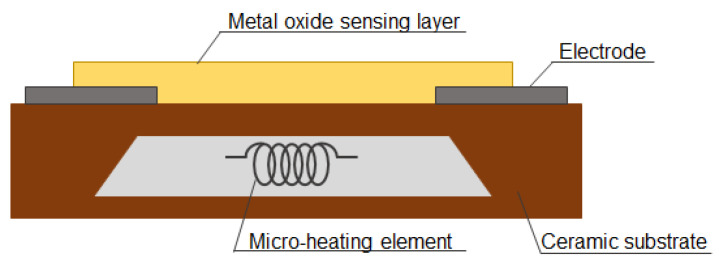
The design of an MOS gas sensor.

MOS gas sensors are based on changes in the physical and chemical properties of the device-sensing material under the influence of gas components undergoing redox reactions on its surface [1]. The key components of a typical MOS gas sensor include the substrate, the metal oxide sensing layer, electrodes, a microheater, and a protective layer [1,8,9]. Figure 1 shows the schematic design of an MOS gas sensor.

The substrate providing a stable and insulating base for the sensor is typically made of ceramic materials such as alumina (Al_2_O_3_) or silicon dioxide (SiO_2_) [1,10]. There is a metal oxide sensing layer on the surface of these materials, which is the core of the MOS gas sensor, as it determines its main parameters as selectivity and sensitivity. The selection of an appropriate sensing material should take into consideration the shape of the exposed surface area available for material–analyte interactions, the number of active sites for the effective and selective binding of the target analyte, the ability to convert the binding action into a detectable signal and mechanical properties ensuring its easy processing [4,11]. The choice of materials for the sensing layer of MOS gas sensors depends also on the specific application and the type of gases being analyzed, as different metal oxides exhibit varying sensitivities to different gases (Table 1). The properties of various metal oxide semiconductors have been studied extensively. The most common MOS gas sensors are based on tin dioxide (SnO_2_). This metal oxide enables the detection of various gases, including carbon monoxide (CO), methane (CH_4_), nitrogen dioxide (NO_2_), and volatile organic compounds (VOCs) [8,12,13]. Other commonly used MOS gas sensors contain such materials as zinc oxide (ZnO), which is particularly sensitive to hydrogen (H_2_) and nitrogen dioxide (NO_2_) [14,15], tungsten dioxide (WO_2_), which is sensitive to a range of gases, including ammonia (NH_3_) and ozone (O_3_) [16], titanium dioxide (TiO_2_) used for such gases as hydrogen, as well as methanol (CH_3_OH) and ethanol (C_2_H_5_OH) [17], and indium oxide (In_2_O_3_) sensitive to such gases like CO and NO_2_ [10]. Other important elements of MOS gas sensors are two electrodes, usually made of noble metals such as platinum (Pt) or gold (Au), which transmit changes in the electrical signal generated through the interaction of the metal oxide sensing layer with the target gas [8,10]. MOS gas sensors also have an integrated micro-heating element, which is electrically separated from the sensing material, and maintains the operating temperature of the sensor [8,9,10]. The sensor is often fitted with a protective layer to increase its durability and protect the sensing layer from environmental factors. The protective layer is typically made of silicon dioxide (SiO_2_) or silicon nitride (Si_3_N_4_).

### 2.2. Principles of Operation

**Figure 2:**
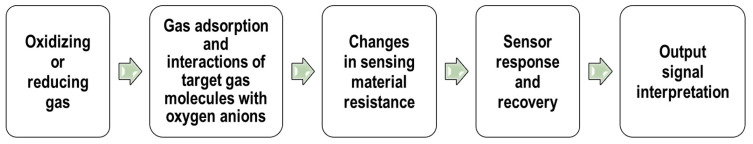
A basic diagram of stages in the operation of MOS gas sensors.

The operation of the MOS gas sensor relies on changes in electrical properties of the semiconductor material when exposed to reducing or oxidizing gases in the surrounding environment [36,37]. In this way, the non-electric chemical information acquired from selective interactions of the target analytes with the sensing material is transformed into an analytically useful and readily measured electrical signal. A basic diagram of stages in the operation of MOS gas sensors is shown in Figure 2.

**Figure 3:**
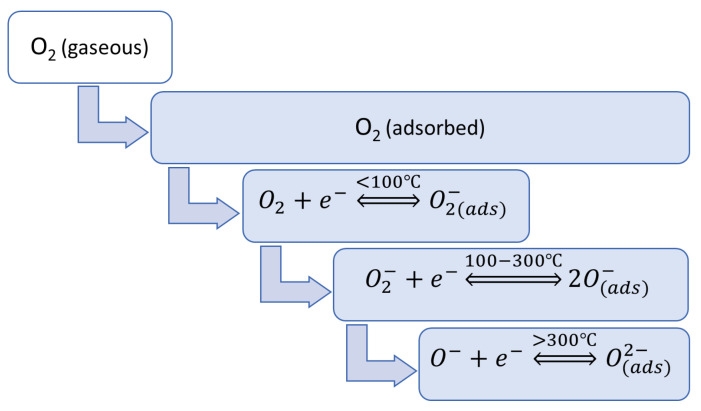
Oxygen adsorption kinematics.

Under typical atmospheric conditions and the operating temperature of a MOS gas sensor, which is usually in the range of 25–500 °C [3], in the absence of the target gas, the sensor semiconductor surface is covered by adsorbed oxygen molecules and characterized by a specific charge distribution [38,39,40]. When the MOS surface layer is heated, oxygen molecules (O_2_) from the air, adsorbed on the metal oxide material, attract electrons from the conduction band and trap them by forming ionic oxygen species such as O_2_^−^, O^−^, and O^2−^ [18,32,41]. As a result, an electron-depleted region, known as the space-charge field, is formed in the sensing material [1,42,43,44]. The type and the amount of adsorbed oxygen ions depends on the working temperature of the sensor, as can be seen in Figure 3 [2,18,32].

When the MOS gas sensor is exposed to a specific gas, its molecules are adsorbed onto the metal oxide surface and an oxidative interaction process occurs between the gas molecules and oxygen anions [45]. The adsorbed gas components can release or accept electrons from the metal oxide surface, changing the concentration of charge carriers (electrons or holes) in the sensing semiconductor material [38,39]. This causes alterations in the depletion region of the active sensing layer and results in variations in the levels of mobile charge carriers [1], which causes relatively substantial changes in sensor resistance. The direction of these alterations (i.e., an increase or a decrease in resistance) depends on the type of gas and the metal oxide material used in the sensor construction.

**Figure 4:**
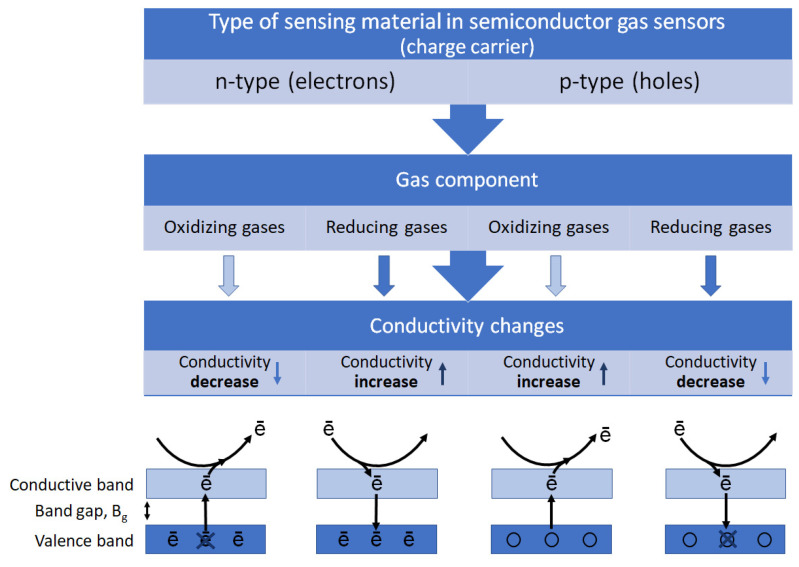
The mechanism of free charge flow and changes in resistance of MOS gas sensors depending on the type of sensor material and analyte.

MOS gas sensors can be divided into two types according to the mechanism of electrical signal transduction, i.e., n-type sensors (based on SnO_2_, TiO_2_, ZnO, WO_3_, In_2_O_3_, MoO_3_, etc.) and p-type sensors (based on CoO, NiO, CuO, Co_3_O_4_, NiO, Mn_3_O_4_, Cr_2_O_3_, etc.) [34,35,46,47,48]. In n-type MOS gas sensors, reducing gases (e.g., CO, H_2_, NH_3_, CH_4_, H_2_S) donate electrons to the semiconductor surface, thus increasing the number of free electrons (charge carriers) in the sensing material, which causes a decrease in metal oxide resistance [2,16,38]. As far as oxidizing gases are concerned (e.g., O_3_, NO_2_), their molecules take electrons from the semiconductor surface. In consequence, the number of free charge carriers is reduced and conductivity decreases [16,40]. The opposite mechanism is observed in p-type MOS gas sensors, where the charge is mainly carried by holes. In these types of sensors, reducing gases deliver electrons to the sensing material and then deplete the hole concentration, and in consequence, the sensor resistance increases. The opposite effect is caused by oxidizing gases [1]. Changes in the electrical conductivity of MOS gas sensors depending on the type of sensing material and target gas are shown in Figure 4.

As a result of above-mentioned processes, the conductivity (and thus resistance, which is more often used in measurement practice) of MOS gas sensors changes as a function of the concentration of the measured gas. Then, the gas concentration is determined on the basis of these changes using various signal processing techniques such as amplifying, filtering, and transforming, which makes the sensor output easier to interpret. Currently, there is no generally accepted definition of gas sensor sensitivity [2]. Usually, assuming that R_a_ is the resistance of gas sensors recorded for the reference gas (typically air) and R_g_ is the resistance observed for the reference gas containing the target gases, sensitivity (S) is designated as a response factor R_a_/R_g_ for reducing gases or as R_g_/R_a_ for oxidizing gases.

The gas sensing process described above involve the chemisorption mechanism, which encompasses the reactions related to adsorption of oxygen and/or target gas molecules on the surface of sensing material. In addition to these chemical actions, a second type of mechanism like physisorption can simultaneously occur during gas detection on the surface of the sensing material. Physical adsorption occurs when gas molecules adhere to MOS crystals through Coulomb forces, hydrogen bonding, and other intermolecular forces, but without undergoing any chemical alterations [49]. Consequently, the change in conductivity of MOS gas sensors caused by pure physical adsorption is negligible. Since chemisorption is usually perceived as the only process accompanied by charge exchange between adsorbed species and metal oxide, physisorption is often overlooked when considering the mechanism of charge flow on the sensor surface in the presence of the target gas [12].

## 3. Advancements in Metal Oxide Gas Sensors

Sensitivity, selectivity, and stability defined as specific interactions between the sensing material and target gas molecules are widely recognized as key parameters of gas sensors [12,50]. Recent research on improving these parameters of MOS gas sensors mainly involves designing new or modified sensing materials [7,50], whereas selectivity enhancement may be additionally achieved thanks to the development of new measurement methodologies [51] and elaboration of new techniques to interpret sensor responses by extracting specific features of the measured signal [5].

### 3.1. Progress in Sensing Materials

**Figure 5:**
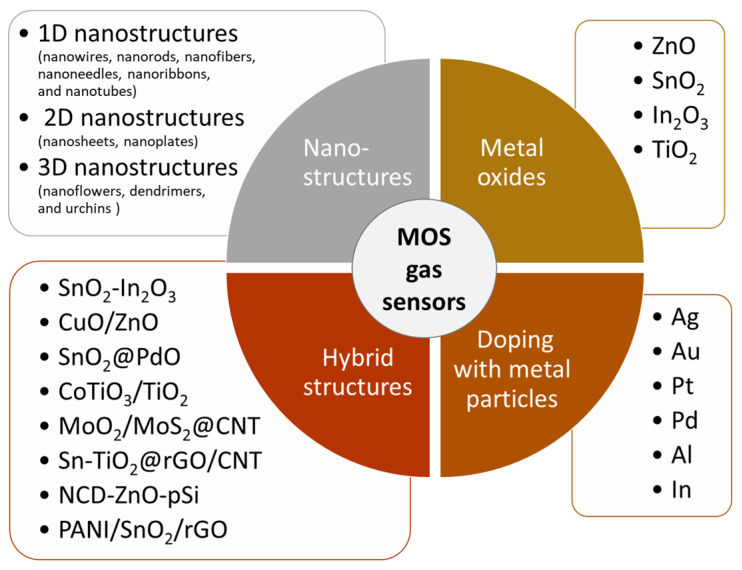
Sensing materials used in MOS gas sensors.

Enhancing adsorption of gas molecules on the sensing surface is crucial for improving gas sensor sensitivity, selectivity, and stability [52]. This can be achieved through several strategies (Figure 5), including increasing the number of available adsorption sites, promoting the formation of oxygen vacancies, and enhancing surface catalytic activity. The following sections of the review present some methods that have recently been used to reach this goal.

#### 3.1.1. Nanostructure Integration

Performance of the MOS gas sensor can be increased using highly porous nanostructures [21]. It was found that MOS gas sensors based on these modern nanoscale materials have significant advantages over traditional ones. A comparison of the response of ZnO dense films (with compact morphology) and ZnO nanoparticles in MOS gas sensors for the detection of H_2_ showed that porous nanostructures play an important role in improving sensor sensitivity [15]. This beneficial effect of nanostructures on the operation of MOS gas sensors has been observed in many studies [20,25,35,53]. The use of metal oxide nanoparticles in gas detection devices improves their performance by reducing response time and increasing sensitivity, especially in the case of low concentrations of gas compounds even in complex and multifarious environments [19,54,55]. The application of nanomaterials exhibits also improved selectivity towards the analyte over interfering gases [19,54].

Research on the sensitivity of MOS gas sensors containing nanostructures have shown that their performance significantly depends on the particle size and morphology of the used nanomaterial [18,19,24,31,56,57]. Advances in nanotechnology have resulted in the incorporation of various types of nanomaterials into gas sensor designs. These include the following: 1D nanostructures in the form of nanowires, nanorods, nanofibers, nanoneedles, nanoribbons, and nanotubes; 2D nanostructures in the form of nanosheets and nanoplates; and also, 3D nanostructures such as nanoflowers, dendrimers, and urchins [3]. Various methods have been developed to enable the fabrication of MOS gas sensors using nanostructures with specific shapes, including chemical vapor deposition [29], the hydrothermal method [26,27,33,53,58], co-precipitation [55,59,60], the sol–gel method [15], pulsed laser deposition (PLD), radio frequency (RF) and sputtering, thermal evaporation [61], spray pyrolysis [62,63,64], and SILAR [65,66]. The advantages resulting from the use of nanostructures are connected with their unique properties, such as a high surface-to-volume ratio, which rises rapidly for small-diameter nanoparticles, improved catalytic activity and tunable morphology, which intensify target gas adsorption onto the sensing material of the sensor [18]. The highly developed surface of these structures taking the core-in-hollow-shell spheres, nanoflakes, nanoplates, or nanosheets made it possible to obtain considerably dispersed catalyst particles that, apart from the enhanced sensing area with a larger number of gas adsorption sites, provide numerous paths for the diffusion and transfer of molecules, electrons, and ions during the adsorption/desorption of oxygen species and target gas [19,31,56,57]. Furthermore, these numerous high-energy sites on the surfaces of metal oxide nanostructures can act as centers for the nucleation and adsorption of noble metals and other materials employed to functionalize the surface and enhance its catalytic activity [67,68,69].

In the literature, we can find numerous examples of the application of MOS gas sensors based on nanomaterials (NMOS gas sensors). It was proved that NMOS gas sensors containing nanoparticles in the form of facet-exposed crystals have excellent sensing properties, which could be attributed to the large number of dangling bonds on their surface [19]. Additionally, nanostructures contribute to the creation of a multitude of various adsorbed oxygen species that can be readily generated through the chemical interaction between the surface and adsorbed oxygen molecules [53]. One-dimensional ZnO nanostructures such as nanotubes, nanorods, nanowires, nanofibers, and certain hierarchical architectures composed of one-dimensional nanostructures exhibit remarkable conductivity [19,35]. Research on the effects of the various ZnO nanostructure morphologies, such as 1D nanofibers, 2D nanoplates, and 3D nanoflowers synthesized via simple electrospinning and hydrothermal routes, on the gas sensing property showed that NMOS gas sensors containing 1D nanofibers showed the highest sensitivity, reversible response, and good selectivity towards formaldehyde (HCHO), as compared with other target gases [25]. The authors attributed these desirable sensing features to the network structure characterized by larger specific surface areas, as well as the one-dimensional arrangement of nanocrystallites with a larger proportion of the depletion layer. Another study has shown successful application of the mixed CuO nanostructure morphology (nanorods, nanoplates, and nanoparticles) for the fabrication of acetaldehyde sensors [35]. The CuO-based NMOS gas sensor showed an excellent dynamic resistance response for various concentrations (20–100 ppm) of acetaldehyde at 180 °C. Moreover, it exhibited high stability and greater selectivity towards acetaldehyde among various gases. The authors indicated that the mixed morphology of the CuO nanocrystalline could have a positive impact on its gas sensing properties thanks to the formation of different interfacial surfaces. It was also revealed that ZnO nanoparticles fabricated in the form of nanoflowers composed of nanoplatelets were more suitable for gas detection than other morphologies thanks to their inherent properties such as surface hierarchical structures and the availability of huge spaces for gas diffusion [21]. Wei et al. [27], while investigating hierarchical WO_3_ nanostructures of nanorods, nanospheres, and nanoflowers synthesized through the hydrothermal route, observed that gas sensing properties of the nanoflowers-based sensor had the highest gas sensing efficiency to acetylene (C_2_H_2_), including a short gas response and recovery time, as well as long-term stability and repeatability. Other research on the effects of crystallinity and size of grains in ZnO nanofibers showed that both parameters competitively influenced the sensing performances of the nanofibers [20]. The study showed that the enhancement of crystallinity plays a dominant role at lower calcination temperatures, whereas at higher calcination temperatures, the grain growth effect became increasingly important due to the marginal enhancement of crystallinity with rapid nanograin growth. This means that in order to achieve a superior performance of these sensing materials based on metal oxide nanofibers, it is necessary to optimize both the size and crystallinity of their grains, as the sensing mechanism implemented during gas detector development can yield a distinct morphology with different dimensions of nanostructures [3,70]. Research focused on testing the properties of metal oxides exposed to highly active factors to assess their potential for use in particularly demanding conditions has also been conducted. An example of this approach is a study by Zdorovets et al. [71] investigating the impact of radiation processes on the structural, optical, and mechanical properties of BeO. During the studies, the kinetics of changes in metal oxide properties due to radiation dose were established, and the critical doses at which it deteriorates significantly were determined.

Sensors based on nanostructures exhibit many advantages, but they also have some drawbacks. One of them is the limitation of the operating temperature of such sensors, which results from the fact that at elevated temperatures small grains of the sensing material tend to agglomerate into large entities, which causes a decrease in the surface areas and catalytic properties of the sensor. Therefore, it is vital to maintain a balance between a reduction in nanograin sizes and sensor stability [2,53]. Moreover, the high operating temperature of NMOS sensors limits their application not only due to the high energy consumption and long-term instability of these sensors, but also due to the potential to produce flammable or explosive gases with a low flash point under atmospheric conditions. Hence, in further work on the design of detection materials, special attention was paid to effective strategies for creating gas sensors operating at room temperature.

#### 3.1.2. Modification of Sensing Material by Noble and Other Metal Particles

The performance of MOS gas sensors can also be enhanced through the manipulation of the catalytic properties of the sensor [3]. It is a common approach to modify the metal oxide material by incorporating a low concentration of noble and transition metal particles, including Pd [72,73,74], Pt [15,75,76], Au [13,74], Ag [58], In [77], Co [59], Al [62,78,79,80], etc., onto the surface of a semiconductor or within its structure. The selection of an appropriate doped element for the sensing material and precise control of the doping element concentration can significantly influence ultimate stability of the gas-sensitive layer. Several methods are available to enrich sensor surfaces or the structure of the sensing material with noble and transition metals. Techniques such as impregnation, sol–gel, sputtering, and thermal evaporation are usually employed to create a composite blend of noble metal particles and metal oxides, while sputtering and thermal evaporation methodologies can be utilized to modify the surface of metal oxides [3]. Recent studies have additionally found that the application of the ion-beam method, combined with several alternating deposition and partial sputtering cycles of a nanosized metal layer, allows nanosized metal films to be obtained that are characterized by stronger adhesion to the substrate compared to those obtained through a single, direct deposition. The authors stated that this may be due to the high energy of the deposited metal atom flux, which upon colliding with the atoms of the substrate and the growing metal layer leads to the formation of nanolayers with better morphological, electrical, and optical properties [81].

There are two concepts that may explain the role of noble metal particles in improving the activity of sensing materials in MOS gas sensors. One of them is the catalytic activity, as noble metal particles deposited onto the surface of semiconductor materials, such as tin dioxide (SnO_2_) or zinc oxide (ZnO), can effectively reduce the activation energy required for gas reactions [21,82]. They facilitate the dissociation of gas molecules into ionic species, such as oxygen ions (O_2_^−^) or oxygen vacancies, which can alter electrical properties of the semiconductor. The supplementation of the sensing material with noble metal particles increases the amount of oxygen species and accelerates the gas sensing process. This results in an enlargement of the depletion layer and, consequently, provides a higher base resistance, which makes the sensor more sensitive and responsive to low concentrations of target gases [21,41]. This improved catalytic activity leads to faster detection and recovery times and it enhances sensor selectivity by promoting specific gas reactions over others. Metal nanoparticles used as catalysts/dopants can efficiently mitigate sensor cross-sensitivity to interfering gases, which is critical for accurate gas detection and quantification [19,58]. An example of such improvement is given in a study in which the integration of Ag particles with ZnO nanorods significantly increases the selectivity of the Ag/ZnO nanorod-based sensor towards NO_2_ gas molecules, as compared with other gases such as SO_2_, methane, CO, ethanol, methanol, NH_3_, H_2_, and formaldehyde [58]. Another advantage of doped metal ions and noble metal particles dispersed on the semiconductor surface is the formation of a larger active surface area [41,58,83]. In consequence, the number of active sites for gas molecule adsorption and reactions increases, while the charge transfer between gas molecules and the semiconductor material is facilitated. Apart from the greater sensitivity of the sensor, the enlarged active surface area resulting from metal doping may also improve the long-term stability and durability of the sensor [15,48].

It is known that the optimal operation temperature of an MOS gas sensor depends on various factors, including the type of target gas, and the chemical composition and morphology of the sensing material [21,27,84]. As mentioned earlier, the operation of an undoped MOS gas detection device at high temperatures limits its use due to increased energy consumption. Moreover, at higher temperatures, their response is restricted by the rate at which gas molecules diffuse [79]; therefore, the most effective response occurs at moderate temperatures, because thermal energy is sufficiently elevated to surpass the activation energy barrier, thereby enhancing reaction kinetics. Within this temperature range, the rates of adsorption and desorption attain an equilibrium, which results in the highest sensitivity [3]. The incorporation of noble metal nanoparticles into the sensing material enhances efficiency of MOS gas sensors at lower temperatures and enables them to detect target gases even at trace concentrations [13]. Research on the application of ZnO nanorods assisted by a Pt catalyst in the MOS gas sensor for H_2_ detection showed that sensitivity of the sensor based on doped ZnO, which is able work at room temperature, was five times greater than that of the sensor based on pure ZnO dense film operating at 200 °C [15]. Another study showed that fabrication of a gas sensor in the form of highly dispersed Au nanoparticles evenly distributed over the surface of the SnO_2_ film also led to the development of a sensitive and selective device for NO_2_ working at room temperature [13]. The authors noted that the designed Au/SnO_2_ sensor exhibited reduced sensitivity to NO_2_ as the temperature increased. This suggested that room temperature was the optimal operating temperature for NO_2_ detection. Moreover, the response of the sensor towards 50 ppm NO_2_ at room temperature was 5.5 and 3.2 times greater than that of the gold-layer-loaded SnO_2_ and pure SnO_2_ sensors, respectively. Similarly, the incorporation of Au elements into ZnO nanorods improved the gas sensing properties compared to those constructed with the used of pure ZnO-based nanorods [74]. The sensor based on Au-doped ZnO nanorods had the highest distinct signal as well as short response and recovery times, high sensitivity with a detection limit of 5 ppb, low operating temperature, and also good long-term stability towards acetone. Gas sensors based on unique nanosized Pt-decorated hierarchical ZnO microspheres composed of porous nanosheets also responded to triethylamine (TEA) gas better than pure ZnO microspheres and Pt-c-ZnO (Pt nanoparticles deposited on commercial ZnO). Apart from improved selectivity, they were characterized by lower working temperature and long-term stability [75]. It is worth emphasizing that lowering sensing temperatures by doping metal oxides with noble metals is an energy-efficient solution, as power consumption of sensors is reduced, whereas their operational lifetime is extended [3]. However, lower operating temperatures may result in longer response and recovery times for these sensors due to the slower rate of kinetic reactions between semiconductors and the target gas [3,85].

#### 3.1.3. Hybrid Structures

As earlier mentioned, a material suitable for gas detection should possess a number of features, such as adsorption/desorption ability, thermodynamic stability, catalytic activity, sensitivity, and the ability to efficiently convert the chemical process into a detectable signal at low temperature. Although various metal oxide-based materials seem promising in terms of some of these properties, few of them fulfill all the requirements simultaneously. Therefore, beyond nanotechnology and metal doping, further improvement in sensor materials focuses on designing more advanced sensor materials (heterostructures), the properties of which are derived from the synergistic effects of combining different components [3].

##### Combination with Other Metal Oxides

**Figure 6:**
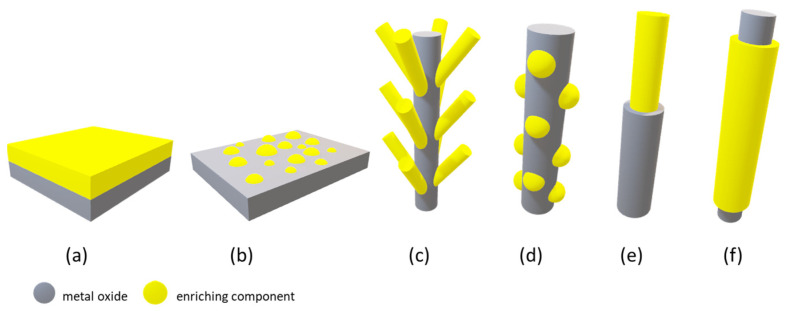
The most common morphologies found in heterostructures composed of metal oxides, i.e., (**a**) bi-layered, (**b**) mixed, (**c**) branch, (**d**) decorated, (**e**) longitudinal, and (**f**) core–shell.

The most basic heterostructure configuration is a composite consisting of two metal oxides [4]. The ways in which composite materials are combined include (1) mixtures of randomly distributed metal oxides (designated as a-b, e.g., SnO_2_-In_2_O_3_), (2) diverse structures, in which there is a clear division or sharp boundary between two or more oxides (designated as a/b, e.g., a core–shell NiO/TiO_2_ nanowire or a two-layer structure), and (3) a base material, on the top of which a second material is added (or loaded) (designated as a@b, e.g., SnO_2_@PdO, in which SnO_2_ may represent nanowires covered with PdO nanoparticles) [7,86]. The characteristics of the hybrid structure result not only from the combination of the physicochemical properties of both of its components, but also from their operating mechanism. Some features that require individual examination also result from the way the materials are integrated [7]. Figure 6 summarizes some of the most common morphologies (i.e., bi-layered, mixed, branch, decorated, longitudinal, core–shell) found in these metal oxide heterostructures. 

Depending on the nature of the semiconductor, adsorption and desorption processes, occurring on the surface of the composite material, lead to alterations in the exchange of charge carriers in local p-n, n-n, and p-p junctions between the two different solid-state materials, with p-p being rarely reported in the literature on the subject [4,7,87]. In the case of very popular p-n junctions, electrons flow through the interface between the n-type and p-type metal oxides, tending to reach the Fermi level equilibrium. In the presence of oxidizing or reducing gas molecules, this electron–hole recombination process leads to greater changes in resistance, contributing to an enhancement of the sensing response, as more carriers participate in the charge transfer. An n-n or p-p junction induces band energy level bending in a manner similar to p-n junctions [7]. At the interface of an n-n junction, there is a straightforward transfer of electrons from the material with a higher Fermi level to the one with a lower Fermi level, resulting in the formation of an accumulation layer rather than a depletion layer. The subsequent adsorption of oxygen on the surface can deplete this accumulation layer, effectively raising the potential energy barrier at the interface and thereby enhancing the sensing response.

Numerous studies on sensors fabricated using metal oxide composites confirmed their enhanced performance [88,89]. For instance, a study on NiO-ZnO hybrid microspheres (p-n heterojunction) showed their better acetone sensing properties compared to a sensor based on a conventional single-component material (pure NiO) [56]. Its potential application in high-performance gas sensors was indicated by a higher value of the response factor (S = 7.5) and shorter response and recovery times (8 s/13 s) at a lower working temperature (260 °C). The authors attributed this increased sensor performance to both the synergistic interaction between p-type NiO and n-type ZnO and the unique structures of double-yolk-shell hybrid components, in which porous shells of hybrid components provide enough active sites to facilitate electron transport during adsorption/desorption processes. Another study also indicated that the formation of p-n junctions in NiO/ZnO heterostructures composed of the cubic structure of NiO and the hexagonal structure of ZnO facilitates the design of sensors that are much more effective in detecting acetone than those based on pure NiO and ZnO, while lowering the operating temperature (from 420 °C for pure NiO to 330 °C in the case of the composite) [87]. In turn, the synergistic effect of high sensor sensitivity resulting from the n-n heterojunction was shown in a study on a novel SnO_2_-TiO_2_ hollow nanostructure consisting of yolk double-shelled microspheres [57]. It has been demonstrated that the sensor based on SnO_2_-TiO_2_ yolk double-shelled microspheres exhibited a high response factor (S = 9.4) and a short response/recovery rate (1.7 s/13.6 s) to ethanol gas. The authors attributed the fast sensor response and recovery to the special multi-shelled hierarchical structure, which facilitates multiple diffusion pathways of oxygen and target gases between the outer and inner spherical shell. Additionally, it was emphasized that structure porosity provides more active sites and improves permeability, facilitating the diffusion and transmission of gas molecules not only across the inner surface, but also the outer surface of the sensor material. Furthermore, the mechanisms of electron transport from TiO_2_ to SnO_2_ through band bending result in the formation of a potential barrier within the heterostructure, thereby significantly increasing the sensor response. 

Further development of the sensory capabilities of heterostructures involves their modification with noble metal particles. These compounds, known for their effective catalytic properties, can be used to improve the reactivity of the sensing material on the gas sensor surfaces [3]. Many previous reports indicated satisfactory effects resulting from the use of noble metals in pure metal oxide-based gas sensors [62,76,90]. In the case of metal oxide heterostructures, improvements in sensor performance associated with noble metal doping were also observed. For instance, it was found that the sensor fabricated using Ag-modified In_2_O_3_/ZnO nanobeams exhibits good formaldehyde recognition ability in terms of a low detection limit of 100 ppb, short response and recovery times (6 s and 3 s), and relatively low operating temperature (100 °C), compared to sensors based on unmodified composites [85]. The authors explained that the excellent gas detection efficiency of the sensor based on the Ag-modified In_2_O_3_/ZnO nanocomposite may result from several factors. One of them is the modification of the composite with Ag that can enhance the electrical conductivity of the sensor, consequently resulting in its increased responsiveness. Moreover, the porous nature of the fabricated heterostructure might increase the number of active sites on the sensing surface to react with gas compounds, thus lowering the detection threshold. The authors also indicated that the porous morphology of Ag-modified In_2_O_3_/ZnO nanobundles can additionally inhibit nanocrystal aggregation when compared to a monodisperse scenario. This attribute proves advantageous by averting the undesirable aggregation of these nanostructures and maintaining high sensor stability. Additionally, the presence of nanogaps in a porous composite structure can enhance gas diffusion rate, which is responsible for shortening reaction and regeneration times. A significant increase in sensitivity in benzene detection was also observed in a study on a sensor based on a Pd-doped CoTiO_3_/TiO_2_ nanocomposite taking the shape of three-dimensional hierarchical flower-like nanospheres [91]. The authors noted that the sensor response exhibited an upward trend corresponding to the increasing complexity of the heterostructures utilized in sensor fabrication, as follows: TiO_2_ < CoTiO_3_/TiO_2_ < Pd doped CoTiO_3_/TiO_2_. They indicated that Pd nanoparticles randomly dispersed on the surface of CoTiO_3_/TiO_2_ nanostructures ensure good contact with this composite, which together with its nanoflower morphology provides a large active surface area for gas adsorption. The benzene detection limit as low as 100 ppb and a high sensor response factor (S = 33.46 at 50 ppm) were attributed by those authors both to the catalytic effect of Pd and the formation of the CoTiO_3_/TiO_2_ p-n heterojunction.

Novel sensors based on the heterostructure synthesized by atomic layer deposition (ALD) also show promising gas sensing behavior. This innovative architecture enables the management of oxygen vacancies, which ensures much better performance in detecting gas molecules at lower operating temperature and exceptional sensing properties, such as higher sensitivity and a lower detection limit [82]. A notable example of this approach is the research conducted by Xie et al. [92], in which they utilized oxygen vacancy engineering to fabricate a gas sensor based on In_2_O_3_/NiO heterostructures. Their innovative approach led to an increased generation of oxygen vacancies, resulting in a decreased resistance of In_2_O_3_/NiO films and subsequently improved NO_2_ sensing performance. The optimized In_2_O_3_/NiO-based gas sensor exhibited outstanding performance, achieving a significantly enhanced response (S > 500) even at a low NO_2_ concentration of 10 ppm while operating at 145 °C. Furthermore, it demonstrated an impressive lower limit of detection and was capable of identifying NO_2_ at levels as low as approximately 6.9 ppb. It seems that the described strategy reported herein will provide an effective and feasible solution for optimizing the performance of other MOS sensors.

The combination of various components with excellent electrical properties may result in new composite materials that can be used to improve the gas detection processes. An example of such material can be a compound based on metal oxides in the form of TeO_2_-(1-x)ZnO-xSm_2_O_3_, which was obtained by Kozlovskiy et al. [93] using a mechanochemical synthesis followed by thermal sintering. The tested material was characterized not only by its usefulness for gas detection properties, but also by its high resistance to aggressive external factors.

##### Application of Carbon-Based Nanomaterials

Carbon nanomaterials such as carbon nanofibers (1D), single- or multi-walled carbon nanotubes (1D), graphene (2D), and carbon quantum dots play a crucial role in generating, amplifying and identifying sensing signals in many areas of application, including environmental monitoring and life sciences [94,95,96,97,98]. The heterocyclic nature of carbon–carbon (C-C) bonds within these nanomaterials governs their characteristic spatial configurations, leading to remarkable chemical and electric properties. Low operating temperatures are provided thanks to the incorporation of highly conductive materials, such as carbon nanotubes, graphene, and other forms of activated carbon into MOS gas sensors [98].

Among carbon nanomaterials, namely nanotubes, nanohorns, nanoonions, and nanodiamonds, carbon nanotubes, being hollow tubular one-dimensional nanomaterials, have emerged as one of the most extensively investigated carbon-based materials [96,99,100,101]. Electrochemical properties exhibited by both single-walled and multi-walled carbon nanotubes, including catalytic activity, stability, electrical conductivity, and biocompatibility, have significant implications for their important role in the development of chemical or biological sensors, particularly in the field of food safety [94,102]. Carbon nanotube properties were used in the construction of a MoO_2_/MoS_2_ heterojunction (MoO_2_/MoS_2_@CNT) for hydrogen detection by Ren et al. [103]. The proposed approach ensured a strong interaction between MoO_2_ and MoS_2_, resulting in excellent electrocatalytic performance of the sensing material, as well as excellent stability. 

In recent years, graphene, an electrically conductive allotrope of carbon with atoms arranged in a repeating hexagonal lattice configuration forming a two-dimensional flat honeycomb-shaped sheet, has gained attention for gas sensor applications [104,105,106]. Its usefulness stems from the fact that this material provides an extended surface area, which can be additionally increased by introducing dopants into its matrix. Graphene also exhibits remarkable electrical conductivity, primarily due to the enhanced electron transfer that predominantly occurs at its edge planes [107]. Another important advantage of graphene from a practical standpoint is its low level of electrical noise, attributed to its exceptionally ordered structure with a small number of disturbances in the crystal lattice (crystal defects) [108]. This characteristic of graphene can help gas sensors to minimize the distorting effect resulting from fluctuations caused by the thermal movement of charges, which may lead to internal noise significantly exceeding the output signal generated by individual molecules. The ability of graphene to form a two-dimensional structure, which is practically entirely exposed to the action of the analyzed compound, also means that even a few charge carriers can cause a measurable change in its properties. Schedin et al. [108] demonstrated that a gas sensor fabricated on the basis of this carbon-based material is able to achieve high sensitivity and the capability to detect even individual gas molecules attaching to or detaching from its surface. Additionally, owing to its highly conjugated network, graphene possesses inherent hydrophobic properties, making it an ideal substrate for facile immobilization of organic molecules [104,109]. Thanks to these properties, graphene is often used to create various heterostructures, as a hybrid based on metal oxide containing this material can significantly enhance electron transfer and thus sensitivity and selectivity toward the target gas [109,110]. A good example of such an improvement is an advanced-structure gas sensor using a tin-titanium dioxide/reduced graphene/carbon nanotube (Sn-TiO_2_@rGO/CNT nanocomposite), which has demonstrated exceptional sensitivity and unmatched levels of selectivity, particularly towards NH_3_ compared to other volatile organic compounds (VOCs) such as toluene, dimethylformamide, acetone, ethanol, methanol, isopropanol, formaldehyde, hydrogen, carbon dioxide, acetylene, and VOCs [110]. Its exceptional selectivity was retained even at room temperature, without discernible impact of humidity in the range of 30–70% RH. The authors explained that the proposed ammonia detection mechanism for the Sn-TiO_2_@rGO/CNT gas sensor results from the formation of p-n heterojunctions. The combination of reduced graphene oxide (rGO) and a binary metal oxide semiconductor (SnO_2_-CuO) has also enabled the production of an NO_2_ sensor with unique properties [111]. The sensor based on the designated SnO_2_-CuO/rGO heterostructure demonstrated a high performance at room temperature. Gas sensing analysis reveals that the response of the SnO_2_-CuO/rGO sensor to 50 ppm NO_2_ was 8–15 times greater than that of rGO-based single MOS sensors (SnO_2_/rGO and CuO/rGO). Moreover, this sensor exhibits outstanding selectivity towards NO_2_, with a response six times higher compared to other inorganic gases, and a low limit of detection at 150 ppb. The authors of this approach attributed these exceptional sensor characteristics to the synergistic effects arising from the nanostructure of the composite and the formation of a p-n heterostructure.

Carbon quantum dots (CDs) represent an innovative category of carbon-based nanomaterials characterized by their zero-dimensional structure, typically featuring sizes smaller than 10 nm [112]. For many years, the interest in carbon quantum dots was mainly connected with their potential application as high-performance supercapacitor devices [113]. Nevertheless, in recent years, this material has been gaining increasing interest and usefulness in various other applications, including gas sensor construction [112,114,115,116]. The interest in carbon quantum dots stemmed from the fact that besides the physicochemical properties, characteristic to carbon-based materials, carbon quantum dots possess other features such as unique electron transfer capabilities and a significant surface area, which provide them with extraordinary electrochemical properties [112,117,118]. The presence of surface functional groups including -OH and -NH_2_ on carbon quantum dots provide favorable anchoring active sites, facilitating the fabrication of multicomponent, high-performance composite materials [117,118,119]. Moreover, the incorporation of heteroatoms (N, S, P, B, etc.) into carbon quantum dots in multicomponent systems of sensing materials increase electron transfer through internal interactions and thus improves their electrocatalytic properties [120]. These strong interfacial interactions in multicomponent nanocomposites also promote intermolecular electron transfer [121], which is a key factor in improving performance of gas sensing materials [122,123]. It has been demonstrated that the introduction of nitrogen-doped carbon dots (NCDs) onto ZnO-coated pSi substrates (NCD-ZnO-pSi hybrid structure) enhances the sensitivity of the hybrid CO_2_ gas sensor. The produced device exhibits improved detection response compared to the undoped ZnO-pSi-based sensor, showing an approximately 37% increase in signal strength at an operating temperature of 200 °C with a response time of <30 s [124]. The enhancement of the sensor performance upon the addition of carbon dots is attributed to the heightened CO_2_-oxygen reactions on the ZnO surface due to an increased density of free electrons at the metal-semiconductor-type junction. The authors emphasized that a significant increase in detection speed (~24%) also at a low operating temperature (100 °C) opens up the possibility of developing highly scalable gas sensors containing carbon dots, characterized by low operational costs, easy production methods, and usage of affordable materials. High selectivity for H_2_S in the presence of other interfering gases at room temperature was also observed for the hierarchical structure composed of graphene quantum dots (GQDs) and SnO_2_ quantum nanoparticles embedded in ZnO nanosheets [125]. The produced gas sensor was characterized by a high response factor (S = 15.9 for 0.1 ppm H_2_S) and short reaction and recovery times (14 s and 13 s). These results can be related to the introduction of a p-n heterojunction and a strong synergistic effect between p-type GQDs and n-type SnO_2_ and ZnO. In turn, Bai et al. [126] successfully prepared hierarchical hybrid architectures consisting of ultrathin 2D flower-shaped MoS_2_ nanoparticles decorated with uniformly distributed SnO_2_ quantum dots (QDs) with a standardized particle size of 2–4 nm. The gas sensor based on this architecture, i.e., SnO_2_ QDs@MoS_2_ used for NH_3_ detection, showed a high value of the response factor of S = 8.6, and short reaction and moderate recovery times (6 s and 121 s) for 100 ppm NH_3_ with good selectivity. Additionally, the sensor had excellent repeatability and exceptional long-term stability at room temperature of 25 °C. The author emphasized that the enhanced performance of the gas sensor primarily stemmed from the synergistic interaction between SnO_2_ quantum dots and MoS_2_ nanosheets.

##### Modification with Two-Dimensional Materials

In recent years, two-dimensional (2D) transition metal dichalcogenides (TMDs) have also gained attention for gas sensing applications. TMDs are inorganic compounds type MX_2_, in which M represents the transition metal element (such as Ti, Zr, Hf, V, Nb, Ta, Mo, W, Tc, or Re in groups IV, V, and VI of the periodic table), and X stands for the chalcogen elements (S, Se, Te) [127]. They characteristics can vary depending on the unique combination of transition metal and chalcogen, resulting in a broad spectrum of materials. As a result, TMDs can manifest in many configurations, including semiconducting, metallic, and superconducting phases [128]. In recent years, progress in thin-film synthesis technologies has facilitated the fabrication of monolayer superconductors (2DSC TMDs) allowing them to be seamlessly integrated with 2D and 3D printing technologies. This enables the creation of wafer-scale systems with enormous technological capabilities [129]. The discussed two-dimensional structures offer outstanding chemo-physical and electric properties including tunable band gap energies and an exceptional charge carrier mobility [130,131,132,133]. These distinctive attributes of TMDs render them well-suited for applications in gas sensing, as alterations in electrical conductivity, stemming from the adsorption of gas molecules onto the material surface, can be leveraged to identify and measure the concentrations of particular gases. The hybridization of MOS with TMD leads to significant improvement in its performance, mainly due to the synergistic effect of the electrical, chemical, and geometric characteristics of the used sensing materials [127]. The integration of these materials results in a notable expansion of the active surface of the sensing material, thereby facilitating selective diffusion and adsorption of particular target gases. Additionally, this hybridization improves the efficiency of charge carrier transport when the device comes into contact with gas molecules [82,134]. Many transition metal disulfides in the form of two-dimensional layered materials such as MoS_2_, WS_2_, SnS_2_, TaS_2_ are excellent representatives of TMDs for gas sensing applications [82,127,131,135]. However, electrical properties of TMDs, such as tunable band gap energies and exceptional charge carrier mobility, are thickness-dependent and increase as the thickness of the semiconductor decreases to one or few layers [127,133,135,136]. The study by Qin et al. [137] on the NH_3_ sensor based on WS_2_ films, ranging from bulk to monolayer, revealed a significant improvement in its sensing response as the thickness of the WS_2_ film decreased. The influence of the layer thickness on the sensor response was also investigated by Zhang et al. [135]. They fabricated a composite sensor comprising MoS_2_ and Co_3_O_4_ with varying numbers of layers on a substrate by the layer-by-layer self-assembly method and noted that the sensor containing the five-layer composite exhibited the most favorable response when exposed to NH_3_ at room temperature. Similarly, CuO/MoS_2_ nanohybrid composites provided impressive results, including high sensitivity, rapid response, and excellent stability in detecting H_2_S gas molecules at room temperature [136]. The authors ascribed the increase in efficiency of gas detection for this composite to the synergistic effects of binary nanostructure and modulation of charge transfer by the formed p-n heterojunction. Another hierarchical nanostructure composed of flower-like SnS_2_ and ultrafine SnO_2_ nanoparticles containing an increased number of active sites for NO_2_ adsorption was tested by Hao et al. [138]. This hybrid structure exhibited an ultrahigh value of response factor S = 51.1 toward 1 ppm of NO_2_ at 100 °C. The flower-like SnS_2_ structure facilitated NO_2_ adsorption and the SnS_2_/SnO_2_ heterojunction played a crucial role in enhancing the sensing response of the device. A recent study also demonstrated the amplifying properties of TMD by examining a flower-shaped hybrid composite of MoO_3_ and MoSe_2_ for the detection of trimethylamine. The MoSe_2_ morphology and heterojunction formation resulted in exceptional gas sensing performance [139]. The manufactured nanocomposite-based sensor was characterized by high sensitivity, low detection limit, fast response, excellent stability, and exceptional selectivity even at room temperature.

##### Conducting Polymers

Another approach to improving sensing materials is to mix metal oxides with conducting polymers including polyacetylene (PA), polyaniline (PANI), polypyrrole (PPy), polythiophene (PT), poly (3,4-ethylene dioxythiophene) (PEDOT), and poly(phenylene vinylene) (PPV) [140]. These materials possessing characteristic π-conjugated structures typically demonstrate p-type conductivity [140,141]. In contact with gas molecules, they can act as either electron donors or acceptors, leading to changes in charge carrier concentration and subsequently affecting the polymer resistance [141]. Conducting polymers have displayed significant promise as active layers in gas sensing applications thanks to their sensitivity to chemical molecules [131,142], flexibility, low cost, ease of synthesis, and excellent mechanical properties [140,141]. The main advantage of these materials in gas sensors based on conducting polymers is that they can operate effectively at room or elevated temperatures [4,131,140]. Nevertheless, these materials also have some limitations. Although the conjugated chains give polymers conductivity, its value is relatively low because of the structural and morphological disorder in the polymer matrix; therefore, in their pristine, undoped state, conducting polymers can be categorized as either electrical insulators or semiconductors [141,143]. Moreover, stability of conducting polymers is particularly sensitive to ambient conditions, including fluctuations in humidity and temperature, which can influence both the chemical and physical properties of the gas-sensing conducting polymer layer [144]. Consequently, sensors employing conducting polymers tend to have a relatively short operational lifespan, as their response tends to diminish with time and this deterioration is not recoverable [4]. Stability of conducting polymers can also be significantly influenced by the interaction with the analyte [4]. Such interactions can induce a swelling effect within the conducting polymer layers, which in turn leads to changes in resistance and impacts the overall sensing performance. Due to this conductive polymer’s sensitivity to humidity and temperature, sensors based on them may require careful calibration and compensation for these disturbing factors. Doping processes through protonation or redox reactions, which effectively extract electrons from the polymer backbone, reduce the disorder in the polymer matrix, and increase their conductivity from an insulating to a metallic regime (above 10^−5^ S∙cm^−1^), have been proposed as a solution enhancing conductivity of polymers [141,143,145]. Further treatments were also used to augment their active surface area and introduce functionalization [140]. Recent work has also attempted to improve the properties of polymer films by using swift heavy ion irradiation. The conducted research showed that this polymer treatment allows the creation of reversible non-covalent π–π conjugation bonds between neighboring chain molecules in polymer films and enhanced the polarization effects resulted from the interactions of neighboring polymer chain molecules [146]. Despite notable progress in conducting polymer sensing capabilities, persistent challenges such as low sensitivity, limited reversibility, and reduced selectivity continue to impede practical implementation conductive polymers in gas sensing applications [141].

In recent years, there has been significant interest in utilizing distinctive characteristics of conductive polymers in conjunction with attributes of metal oxides used in MOS gas sensors [142,147,148,149,150,151]. Metal oxide-based sensors are known for their consistently high sensitivity attributed to oxygen stoichiometry and active surface area [1,2,3]. However, their practical application has been limited due to the requirement for high operating temperatures [147]. When searching for new, efficient gas detection devices, researchers hypothesized that the synergistic effect between the properties of conductive polymers and metal oxides could lead to the development of sensors in which a balance between operating temperature and sensor response could be achieved. Research conducted in this area confirmed that this assumption represents a promising avenue for improving gas sensors. The investigations conducted on pure PANI and PANI/TiO_2_ nanocomposite thin films revealed disparities, including not only gas sensing properties, but also surface morphology [152]. Further investigations concerning a hybrid heterostructure of PANI and SnO_2_ developed via polymerization showed that the sensor based on this hybrid material not only exhibits high sensitivity, outstanding selectivity, and a wide linear response to NH_3_ at a room temperature (21 °C), but also possesses a flexible, simple structure, and wearable performance [149]. The authors emphasized that the enhancement of sensing properties can be ascribed to the synergistic and complementary interactions between SnO_2_ nanoparticles and PANI, as well as the establishment of a p-n heterojunction at the hybrid interface. Similarly, the sensing mechanism of PANI/SnO_2_ nanocomposites for hydrogen gas was attributed to the formation of a p-n heterojunction resulting from the combination of p-type (PANI) and n-type (SnO_2_) semiconductors [147]. It was observed that the PANI/SnO_2_ nanocomposite effectively overcame the limitations of pure PANI and pure SnO_2_, displaying increased sensitivity, faster response times, and shorter response-recovery durations at room temperature. As a result, when exposed to H_2_, the PANI/SnO_2_ nanocomposite demonstrated exceptional sensitivity, achieving a rapid response within 11 s and a swift recovery within 7 s, all at room temperature. Enhanced sensor responses were also obtained through by using PANI/SnO_2_ nanocomposites for carbon dioxide (CO_2_) monitoring [153]. This handcrafted sensor displayed repeatability, reliability, and selectivity in its response to varying CO_2_ levels. Additionally, it was observed that the sensor response was contingent upon the tin dioxide (SnO_2_) nanoparticles content in the composite structure. This dependence is also ascribed to the formation of a p-n junction between the PANI chains and SnO_2_ grains, which likely enables inter-particle electron migration from SnO_2_ to the polyaniline chains. As a result, holes are transported both within the chains and between chains, with electron hopping occurring in the SnO_2_ region [154,155]. The effect of metal oxide content in the polymer matrix was also observed in another study, in which the gas sensing results, recorded by the PANI-WO_3_ hybrid nanocomposite sensor synthesized through the polymerization route, displayed improved selectivity towards NH_3_ gas in comparison to PANI and WO_3_ sensors [150]. The higher content of WO_3_ within the PANI matrix resulted in an increased sensor response, primarily attributed to the enhanced porosity of the composite morphology. The above-mentioned observations showed that flexible films of the PANI-WO_3_ hybrid, containing 50% of the metal oxide, exhibited a high response and maintained a stability of 83% when exposed to 100 ppm NH_3_ gas at room temperature. The study, which investigated the impact of the incorporation of the SnO_2_ and rGO on gas sensing properties of PANI nanofibers confirms synergistic interactions between PANI nanofibers and SnO_2_ nanoparticles, as well as rGO nanosheets [156]. In comparison to pure PANI, all PANI/SnO_2_ and PANI/SnO_2_/rGO nanocomposites exhibited superior selectivity and sensitivity towards NH_3_ gas. Notably, the PANI/SnO_2_/rGO nanocomposite displayed the highest response to a 10 ppm concentration of NH_3_ gas at room temperature, with a recovery time of approximately 80 s. During the study, it was also noted that an increase in the concentration of the dopant in the PANI/SnO_2_ nanocomposite resulted in an enhanced response. One of the recent studies presents also a new approach focused on designing a composite based on electrochemically active polypyrrole doped with dodecylbenzene sulfonic acid (PPy-DBSA) and Co-based Y-type hexaferrite [157]. According to the authors, this novel material (the PPY/DBSA + Sr_2_Co_2_Fe_12_O_22_), synthesized using the microemulsion technique and characterized by chemical stability, hardness, exceptional corrosion resistance, and wear resistance, constitutes a promising candidate for practical applications. Research to date shows that the combination of the unique and adjustable electrical properties of conducting polymers with metal oxide features facilitates the development of MOS gas sensors. These sensors, on the one hand, display robustness, long-term stability, and maintain consistent and reliable performance over extended periods, while on the other hand, they are able to selectively detect target gases, while remaining inert to other gas compounds at low operating temperatures [140].

### 3.2. Sensor Thermal Modulation

**Figure 7:**
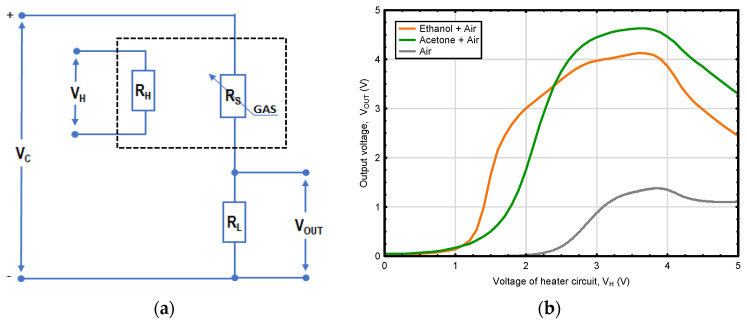
(**a**) The diagram illustrates the measuring system for the TGS2610-C sensor with temperature modulation, with R_S_ representing the sensor resistance, R_L_ denoting the resistance of an auxiliary resistor, V_C_ and V_H_ indicating the voltages of the sensor circuit and micro-heater circuit, and V_OUT_ signifying the voltage associated with the sensor resistance R_S_ [5]; (**b**) the voltage output signal of the TGS2610-C gas sensor, with temperature modulation examined for pure air and of individual analytes: acetone and ethanol with a content of 0.5% [5].

**Figure 8:**
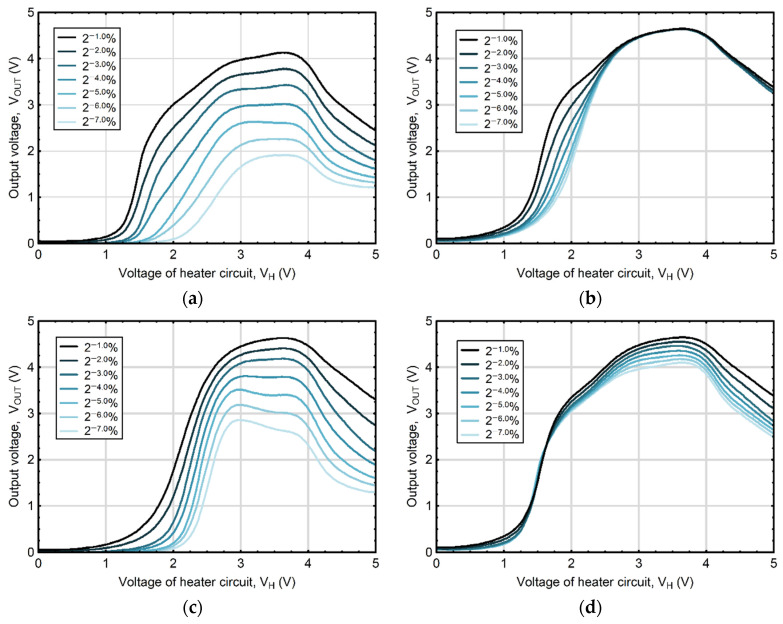
(**a**) V_OUT_ waveforms, showing the response voltage of the TGS2610-C gas sensor with temperature modulation along with linear increasing in the voltage of the micro-heater at rates of 0.5 V per minute, recorded separately for different concentrations of (**a**) ethanol alone, (**b**) ethanol in presence of one level of acetone (0.5%), (**c**) acetone alone, and (**d**) acetone in presence of one level of ethanol (0.5%) [5].

Despite considerable efforts made to improve MOS gas sensors by sensing material modification (Table 2), the widespread application of these devices is still hampered by their constrained selectivity [110]. Therefore, in order to enhance their performance, ongoing research and development also focused on optimizing measurement methodologies for MOS gas sensors [158]. The starting point for these considerations is the estimate of the optimal operating temperatures of the gas sensor for various gas molecules. Previous investigations showed that for different gas components, the maximum level of voltage response is often achieved at different sensor operating temperatures. Research by Karmakar et al. [159], examining a gas sensor based on nanocrystalline particles of barium hexaferrite (BaFe_12_O_19_), a material with excellent magnetic properties and stable crystalline structures regardless of temperature [160], showed that the sensor exhibited the maximum response to acetone and ethanol vapors at different temperatures of 325 °C and 375 °C, respectively. As a result, in recent years a promising approach to enhance selectivity of MOS gas sensors has emerged through the development of a measurement technique involving thermal modulation of sensor heater [5]. This advancement opens up promising possibilities for applications that require the precise detection of individual components within gas mixtures [6,161]. The thermal modulation approach turns in the adjustment of input voltage applied to the gas sensor heater, which dynamically changes the sensor operating temperature, offering valuable insights into the kinetics of surface processes [162,163]. In a measurement methodology using this modulation technique, the responses of a single MOS gas sensor can be likened to the action of multiple gas sensors of the same type operating at different temperature levels. Temperature fluctuations modify the intensity of electron transitions from the forbidden to the allowed band and affect adsorption and redox reactions occurring on the surface of the sensing material, thus influencing sensor resistance (R_s_) (Figure 7a). Changes in this resistance, recorded as a function of heater voltage V_H_, create comprehensive characteristics (Figure 7b and Figure 8), providing complex insight into the composition of the mixtures under examination [5]. Moreover, the variation in the waveforms recorded for gas mixtures can be treated as a “fingerprint” containing encoded information about the type and concentrations of its gas components (Figure 8).

Previous research has shown that the interpretation of the response signal recorded by a thermally modulated sensor can be helpful in the quantitative analysis of molecules present in the mixture. Shi et al. [164] obtained high-quality and -quantity recognition of four alcohol homolog gases (100% accuracy) using a dynamic measurement method with ZnO-based MOS gas sensors. Similarly, Krivetskiy et al. [158], using the above-mentioned measurement technique, observed an improved discrimination of chemically related gases, i.e., methane and propane, within a concentration range of 40 to 200 ppm. They accomplished this result under varying real-world atmospheric conditions by employing metal oxide gas sensors based on nanocrystalline SnO_2_ modified with Au and Pd that were subjected to temperature modulation. The authors indicated that a single-sensor thermally modulated gas detection device can be considered as a potential replacement for array-based electronic nose systems in many applications. In another study, Bora and Sarma [165] carried out research, in which they attempted to enhance selectivity of MOS gas sensors by altering the sensor surface temperature. For this purpose, the authors designed a temperature modulation circuit based on pulse width modulation (PWM) to control and adjust the temperature of MOS gas sensors. This resulted in varied signature response patterns from the sensors, which can be harnessed to differentiate between individual gases.

The response of the thermally modulated gas sensor contains information on the composition of the analyzed gas mixture; however, it takes unique, complex patterns in the form of waveforms; therefore, their interpretation requires the use of relevant signal processing techniques. Among the methods used to recognize the response patterns, and hence classify substances contained in the analyzed gas mixture, principal component analysis (PCA) [161,163,166] or partial least squares (PLS) regression [167] are often applied. In some cases, PCA or linear discriminant analysis (LDA) are used to recognize the output signal in combination with other machine learning methods, such as k-nearest neighbor (KNN), logistic regression (LR), support vector machine (SVM), or the random forest algorithm (RF) [162,164,168,169]. Since the interpretation of output signals of thermally modulated MOS gas sensors is a key step determining the detection quality and significance of the obtained results, considerable attention has been paid to the development of algorithms aimed at deciphering sensor response patterns. The study by Krivetskiy et al. [158] demonstrated that employing statistical shape space pre-processing on temperature-modulated signals from metal oxide gas sensors leads to an enhanced ability to identify gases. This improvement is particularly notable when using an artificial neural network (ANN)-based machine learning algorithm, surpassing the performance of previously reported signal processing techniques, such as principal component analysis (PCA), discrete wavelet transforms (DWT), polynomial curve fitting, and data normalization. Recently, the concept of integrating signal processing with nonlinear calibration and machine learning techniques has gained importance and is seen as a promising path to improve selectivity and accuracy of detection in real atmospheric conditions [158,170,171,172,173]. This approach was adopted in a study aimed at developing a methodology for measuring and processing the output signal from a thermally modulated MOS gas sensor, using a B-spline curve and artificial neural networks (ANNs) [5]. The proposed two-stage methodology facilitated quantitative analysis of volatile components present in mixtures containing ethanol and acetone.

In the initial phase, B-spline, which is a function described piecewise by polynomials and approximates complex dependencies, was used to extract relevant information from the gas sensor output signals, taking the form of complex, unique patterns (Figure 8). In the second stage, parameters being the control points shaping the B-spline curve were used as an input vector to the ANN model with a multilayer perceptron structure. The results showed usefulness of combining the B-spline (which effectively reduced the size of the measurement data set while retaining its most important features) and ANN modelling techniques to enhance response selectivity from a thermally modulated MOS gas sensor. The considered approach showed the possibility of extending the potential applications of single, thermally modulated gas sensors in the quantitative analysis of gas mixtures.

### 3.3. MOS Gas Sensor Arrays

The measurement accuracy and selectivity of gas sensors exposed to a multi-component gaseous environment can also be enhanced through the development of devices inspired by the human olfactory system, the so-called electronic noses (E-noses). The E-nose design includes two fundamental elements: (1) the sensor chamber containing the olfactory element in the form of an array comprising various odor detectors, which can respond to a wide variety of chemical molecules, and (2) the output unit equipped with a signal transformation system containing pattern recognition algorithms [51,174]. Similarly, as in the case of a single sensor, the main principle of E-nose operation is the oxidation-reduction reaction of gas on the surface of sensors. Each sensor in the E-nose array responds differently to various gases, and the collective response pattern provides a unique “fingerprint” for the mixture of gas components [175]. Then, once the initial electrical signal is recorded, in further steps, it is transformed using statistical data analysis and pattern recognition algorithms, which play a crucial role in decoding the signal, as well as recognizing and classifying data information. As E-nose responses have the multidimensional nature, data analysis necessitates the use of advanced statistical methods. Among them, the most commonly used are the principal component analysis (PCA) [176,177,178], linear discriminant analysis (LDA) [168,179], partial least squares discriminant analysis (PLS-DA) [167,170], and *k*-nearest neighbor (KNN) [180,181,182]. The statistical analysis is usually supported by machine learning algorithms utilizing random forest [170,172], support vector machines (SVM) [180,182,183], artificial neural networks (ANN) [170,172,184], and other artificial intelligence (AI) methods [181,185].

The electronic nose technique uses various types of sensors, including those based on metal oxides, semiconductive polymers [51], quartz crystal microbalances [186], and surface acoustic wave to detect and identify odors [179]. Among them, MOS gas sensors are widely used in E-nose applications thanks to their numerous advantages, including high sensitivity, short response time, portability, accuracy, stability and durability, a broad response range, and low cost of manufacturing [51,175]. Despite their many advantages, MOS gas sensors also have some disadvantages, and one of the main ones is limited selectivity [177]. Therefore, the incorporation of a group of various types of MOS gas sensors in the E-nose array, combined with significant advancements in sensor technology make it possible to enhance its sensitivity and selectivity in odor detection. Additionally, the integration of tools utilizing modern data analysis techniques has boosted precision and efficiency in interpreting sensor responses, thus improving its accuracy. However, like any technology, E-nose gas detection is not without some drawbacks. The primary mode of E-nose operation involves recognizing the entire gas mixture as a whole, rather than isolating individual gases within the mixture [51]. As a result, even though the E-nose device is able to detect changes in the spectrum of volatile organic compounds (VOCs) and thus discriminate between classes or types of the tested sample, it is not capable of identifying specific chemical compounds, which is a significant limitation when detailed gas analysis is required [177,180,182]. Moreover, VOCs constitute a numerous group; therefore, the design of an E-nose sensor array optimized for one application may not exhibit optimal performance when dealing with odors or gases present in other applications [177]; hence, the design of these devices should be customized to particular applications and target gas compounds.

Despite its inherent limitations, the electronic nose technology has found wide applications in various sectors, including agriculture and forestry [51,167,169,184,187], environmental pollutants detection [181] and medicine [183,188]. The application of E-nose systems is particularly common in food industry [168,170,172,176,189,190,191]. In the study by Teixeira et al. [179], an E-nose consisting of nine SnO_2_-based metal oxide sensors (TGS) proved to be a useful tool in classifying extra virgin olive oils according to the fruity intensity commercial grade. The olfactory sensor device employed in this study successfully detected various volatile chemicals responsible for the positive (e.g., fatty, floral, fruit, grass, green and green leaves attributes) and negative sensory attributes (e.g., sour and vinegary defects) of olive oil. The satisfactorily distinguished attributes of examined oils based on the perceived primary olfactory sensation and its intensity was carried out using both unsupervised techniques (principal component analysis) and supervised methods (linear discriminant analysis). Another study examining the applications of two E-noses adjusted for identifying synthetic flavors revealed that the designed arrays of MOS gas sensors can effectively respond to synthetic aromas [177]. However, it was found that the used gas sensors had some performance shortcomings, as SnO_2_-based MQ sensors proved unstable in testing larger numbers of samples and showed inconsistent responses with each iteration, while SnO_2_-based TGS sensors produced a weak signal. The authors of the study suggested that the unstable response of MQ gas sensors could be caused by the temperature and humidity variations within the sensor chamber, the intensity of the sample aroma, and the unstable velocity of the carrier gas.

Recognizing and classifying response patterns are critical elements in the operation of a multisensor arrays; therefore, there is extensive research aimed at investigating discrimination techniques that can improve efficiency of gas detection systems. Xu et al. [192] proposed a novel hybrid method for gas identification and its concentration detection, which was validated in modeled systems using CO and CH_4_ samples. This approach used kernel principal component analysis (KPCA) to extract nonlinear characteristics of gas mixtures of various components. Subsequently, the recognition of the target gas was accomplished through the application of the K-nearest neighbor classification algorithm (KNN). Moreover, the method incorporates a multivariable relevance vector machine (MVRVM) to perform regression on the multi-input nonlinear signal, thereby detecting mixed gas concentration. Experimental results showed that the accuracy of the proposed method (98.33%) was better than the accuracy of principal component analysis (PCA) (92.50) and independent component analysis (ICA) (84.17%). However, the authors pointed out the need for further research on the identification and detection of multiple gas mixtures. In another study, in which an E-nose comprising seven MOS gas sensors was developed, different techniques for its response discrimination were applied, with the aim of identifying sources of milk (variety of dairy farms) and estimating milk quality indicators, specifically milk fat and protein contents [168]. To identify the milk sources, the study leveraged features extracted from milk odor data obtained from the E-nose, as well as composition features derived from dairy herd improvement (DHI) analytical data. These features were subjected to principal component analysis (PCA) and linear discriminant analysis (LDA) for the purpose of dimension reduction. Subsequently, three machine learning algorithms, namely logistic regression (LR), support vector machine (SVM), and random forest (RF), were used to construct a classification model for milk source (dairy farm) identification. The results of that study indicated that the SVM model, based on fusion features post LDA, demonstrated the best performance, achieving an accuracy of 95%. Additionally, based on the E-nose, extracted features estimation models for milk fat and protein contents were developed using gradient boosting decision tree (GBDT), extreme gradient boosting (XGBoost), and random forest (RF) techniques. The outcomes indicated that the RF models outperformed the others, providing R^2^ of 0.9399 for milk fat and R^2^ of 0.9301 for milk protein. In turn, Liu et al. [170] used four popular machine learning algorithms, namely extreme gradient boosting (XGBoost), random forest (RF), support vector machine (SVM), and backpropagation neural network (BPNN), to distinguish odors of different wines recorded using a portable E-nose with TGS MOS gas sensors. The obtained results showed that among the methods under examination, the BPNN exhibited the most outstanding performance with accuracy rates of 94% in identifying production areas and 92.5% in classifying varietals. In another study, Jiang et al. [181] compared the EQBC-RBFNN technique (i.e., query by committee for radial basis function neural network), which followed human learning mechanisms, with various previously employed discriminant methods (LDA, BPNN, RBFNN, KNN, SVM) in the interpretation of responses of an E-nose equipped with SnO_2_-based MOS gas sensors (TGS) in the detection of three types of indoor pollutant gases (benzene (C_6_H_6_), toluene (C_7_H_8_), and formaldehyde (CH_2_O)). Data processing results proved that the EQBC-RBFNN technique improved the accuracy of E-nose classification.

Recent research shows that integrating thermally modulated MOS gas sensors into E-nose arrays can also improve detection efficiency of these systems. Such an effect was observed in a study by Machungo et al. [180], in which contamination of maize varieties naturally and artificially infected with *Aspergillus flavus* was monitored using a portable electronic nose system containing a sensor array equipped with twelve n-type metal oxide gas sensors thermally cycled over a temperature range of 260 °C to 340 °C. The used array included three palladium-doped sensors (SnO_2_-Pd), three platinum-doped sensors (SnO_2_-Pt), one silver-doped sensor (SnO_2_-Ag), one copper-doped sensor (SnO_2_-Cu), three undoped WO_3_ sensors and an experimental sensor (Extype 1) with undisclosed manufacturer specifications. Interestingly, this sensor system has demonstrated the ability to detect a broad spectrum of volatile compounds, encompassing alkanes, alkenes, alkynes, alcohols, aldehydes, amines, mercaptans, partially halogenated hydrocarbons, volatile acids, and volatile aromatic compounds, with the lower detection limit of 1 part per million (ppm). The used electronic nose system exhibited significant ability to distinguish between aflatoxin-contaminated and uncontaminated samples, correctly recognizing 70% of them. The authors suggested that this measurement methodology could facilitate the initial selection of large numbers of samples and thus reduce their number for further, costly and time-consuming quantitative analyses. In a subsequent study, the same authors compared performance of three E-nose instruments, namely MOS gas sensors (Fox 3000), conductive polymer sensors (Cyranose 320), and thermally cycled metal oxide-doped semiconductor sensors (DiagNose) in the detection of volatile compounds characteristic to aflatoxin-contaminated corn naturally infected with *A. flavus* [182]. Cross-validated classification accuracy of the tested E-nose systems revealed that an E-nose equipped with doped MOS gas sensors with thermocycling was the most effective in detecting aflatoxin contamination in corn (accuracy ranged in 81–94% for DiagNose, 76 –79% for Fox 3000 and 68–75% for Cyranose).

Recent research shows that E-noses have proven to be valuable tools in various applications in odor detection and gas analysis; however, there is still a need to improve their performance that can be enhanced by selecting an appropriate pattern recognition algorithm and application of a new measurement methodology using thermal modulation of MOS gas sensors.

## 4. Application of MOS Gas Sensors in the Food Industry

**Figure 9:**
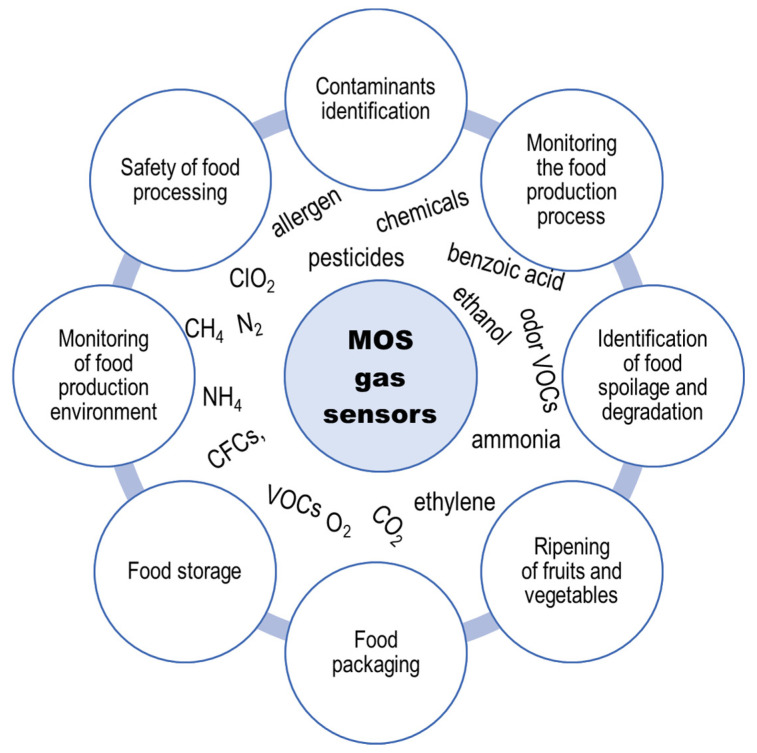
MOS gas sensor applications in food industry.

Figure 9 depicts the most common application of MOS gas sensors in the food industry. The use of a MOS gas sensor is crucial to ensuring safety and quality of food products at various stages of its production and distribution. Apart from that, it facilitates the monitoring of compliance with regulatory standards, as it enables the identification of contaminants such as pesticides, chemicals, and allergens in food products. Research on the detection of two pesticides, i.e., cypermethrin and chlorpyrifos, in apple samples, using an E-nose equipped with ten MOS gas sensors supported by discrimination algorithms, including principal component analysis (PCA), linear discriminant analysis (LDA), and support vector machine (SVM), showed that the employed set of MOS gas detectors accurately identified pesticide residues in apple samples [193].

Gas detection is also essential in the production of various food products like coffee [194], beer [172], cheese [195], and wine [170,196], as well as baking processes [197], in which monitoring the presence of various gases allows the assessment of correctness of production processes and product quality. Experimental results show that the developed E-nose system using SnO_2_-based MOS gas sensors combined with six machine learning techniques (decision tree, random forest, XGBoost, SVM, convolutional neural network (CNN), and CNN+LSTM) achieves good performance in coffee aroma recognition [194]. In the beverage industry MOS gas sensors are employed to monitor the quality of carbonated drinks and ensure consistent levels of CO_2_ [16]. In turn, the application of E-nose technology, in conjunction with ANN modeling, enables both qualitative and quantitative analysis of benzoic acid in cola-type carbonated beverages [198]. These types of gas sensors proved to be the most suitable in classifying different beverages based on the level of ethanol or other attributes [170,172]. For example, an E-nose prototype containing nine QM and four TGS SnO_2_-based MOS gas sensors combined with different regression methods (multiple linear (MLR) and non-linear regression methods (NMLR)), and machine learning techniques (RF, extreme learning machine (ELM) and ANN) were found to be effective tools for detecting the level of ethanol in the range of 4–8% in a high spectrum of beers (for ELV R^2^ = 0.888) [172]. Similar MOS gas sensors (six TGS SnO_2_-based gas sensors) were used in the E-nose bionic system to detect the odors of various wines, followed by four machine learning algorithms (extreme gradient boosting (XGBoost), random forest (RF), support vector machine (SVM), and backpropagation neural network (BPNN)), which, based on the properties of wine, identified it in terms of production area, variety, vintage, and fermentation processes [170]. The best results in identifying production areas and varietals were achieved by BPNN, while the best results in identifying vintages and fermentation processes were delivered by SVM.

Maintaining the right gas composition is critical to preserving the freshness and taste of food. In such applications, MOS gas sensors are used in the fruit and vegetable industry to monitor ethylene produced during ripening in order to control this process and extend shelf life of these products. Tyagi et al. [184] developed an E-nose containing five SnO_2_-based MOS gas sensors and one digital temperature and humidity (DHT) sensor to monitor fruit ripeness. The operation of the MOS sensors incorporated into a microcontroller board was supported by an artificial neural network (ANN) algorithm used for pattern recognition. The designed E-nose system demonstrated the capability to discriminate alterations in odor profiles during the ripening of bananas, apples, grapes, oranges, and pomegranates, successfully classifying these specific fruits into their respective ripeness categories with an average accuracy of 95%.

The use of gas sensors in monitoring the levels of oxygen (O_2_) and CO_2_ in food, such as modified atmosphere packaging (MAP), also helps to extend the shelf life of perishable products by controlling oxidation and microbial growth [199]. In turn, the detection of specific volatile compounds, such as ammonia, ethylene, and VOCs, produced e.g., in the meat and seafood or agricultural industry can help identify the onset of spoilage or degradation [200].

The food industry is subject to various regulations and standards related to food production environment. Gas detection systems can be applied to help food manufacturers comply with these regulations by ensuring that processing environments are free from harmful gases and contaminants. In this area, MOS gas devices found application in monitoring the presence of hazardous gases, such as CO_2_, CO, CH_4_, and NH_3_, which can be released during food processing, storage, and transportation [199]. Some food processing operations may also generate gases, such as greenhouse gases (e.g., methane (CH_4_)) or ozone-depleting substances (e.g., chlorofluorocarbons (CFCs)) that can be harmful to the environment [40]. According to the report of the Intergovernmental Panel on Climate Change (IPCC), food systems account for 23–42% of global greenhouse gas emissions [201]. Therefore, MOS gas sensors can help in monitoring and controlling emissions and thus minimize the environmental impact of food production [202].

MOS gas sensors can also be used to ensure that the concentration of sanitizing gases, such as chlorine dioxide (ClO_2_), is within the required range to effectively disinfect food processing equipment and surfaces. In the case of specialty gases, such as nitrogen (N_2_) used in food processing for cryogenic freezing, gas detectors ensure purity and consistency of these specialty gases, which are critical to the quality and safety of the final product. Gas detection systems often come with data logging and reporting capabilities that allow food manufacturers to maintain records of gas levels over time, enabling traceability and facilitating quality control audits. In the case of a gas leak or abnormal gas levels, gas detectors provide early warning systems that can trigger alarms and shut down equipment, preventing accidents and allowing for a prompt response and evacuation if necessary [203].

## 5. Summary and Outlook

The advancements in enhancing the selectivity of MOS gas sensors hold great promise for the food industry. Gas sensors based on pure metal oxide have exhibited limited selectivity, but recent innovations in MOS gas sensor technology represent a significant step forward in improving the effectiveness of these gas detection devices. One progressive strategy involves the chemical modification of sensing materials. The incorporation of nanostructures and the modification of sensing materials through the integration of metal oxide with metal particles or other elements have a substantial impact on MOS gas sensors, influencing their performance and capabilities. This strategy promotes the creation of a larger active surface area, intensifying the adsorption of target gases onto the sensing material, and increasing the sensor selectivity towards the analyte, while reducing interference from other gases. Chemical doping of the sensing material also improves its electrical properties by altering charge carrier concentrations, thus enhancing sensor sensitivity to specific gases and extending its long-term stability and durability. The combination of the unique properties of doping materials with the metal oxide features of the sensing material also enables the development of MOS gas sensors that can effectively operate at room or elevated temperatures. Another approach that can enhance the performance of MOS gas sensors involves advancements in measurement methodology and interpretation of its response signals. Thermal modulation of MOS gas sensors that causes their operation to be comparable to that of several sensors of the same type operating at different temperatures is good example of such approach. Since the response patterns in such gas detection processes generate complex waveforms, to enhance the quantity analysis of mixed gases, algorithms that combine statistical methods with modern machine learning techniques are continuously developed. Improving gas detection measurements using MOS gas sensors can also be achieved through the development of electric noses equipped with a sensor array design. Combining the properties of several gas sensors allows a reduction in their limitations in the quantitative analysis of gas molecules in a mixture and results in improved recognition capabilities. Advances in MOS gas sensor technology discussed in this review have demonstrated that (a) by reducing cross-sensitivity to interfering gases, (b) lowering detection limits, and (c) providing more accurate data, gas detection has the potential to revolutionize food quality control, safety, and traceability. With the capability to detect spoilage, contamination, and other critical factors in real-time, MOS gas sensors can contribute to extending the shelf life, reducing food waste, and ultimately ensuring that consumers receive high-quality, safe food products at reasonable price. With the enhanced selectivity of MOS gas sensors, the future of food safety and quality control certainly appears promising. Further advancements in sensitivity of MOS gas sensors, especially those utilizing thermal modulation of the heater to monitor gaseous components, may lead to improved performance in the better identification of compounds within gas mixtures ultimately benefiting both the industry and consumers.

## Figures and Tables

**Table 1:** Summary of metal oxides commonly used in MOS gas sensors.

Metal Oxide	Gas Sensitivity	References
SnO_2_	H_2_	[18]
	CO	[12]
	NO_2_	[13]
	methanol	[8]
	ethanol	[19]
ZnO	H_2_	[15]
	CO	[20]
	NO_2_	[21]
	NH_3_	[21]
	methane	[22,23]
	methanol	[21,24]
	formaldehyde	[25]
WO_2_	O_3_	[12]
	NO,	[16]
	NO_2_	[26]
	NH_3_	[12]
	acetylene	[27]
	ethanol	[28]
	toluene	[29]
TiO_2_	methanol	[17]
	ethanol	[17]
	acetone	[30]
In_2_O_3_	CO	[31]
	NO_2_	[10,32]
CuO	H_2_S	[33]
	NH_3_	[34]
	acetaldehyde	[35]

**Table 2:** Summary of advancement in sensing material using in MOS gas sensors.

Sensing Material and Morphology	Target Gas	Synthesis Method	Operating Temperature (°C)	Concentration Measurement Range (ppm)	Response/Recovery Time (s/s)	References
**Nanostructures integation**						
SnO_2_ nanoparticles	ethanol	hydrothermal	200	5–100	9/111 (100 ppm)	[19]
SnO_2_ core–shell nanospheres	ethanol	hydrothermal	260	5–100	1/95 (100 ppm)	[19]
SnO_2_ single nanocrystals	ethanol	hydrothermal	200	5–100	1/15 (100 ppm)	[19]
CuO nanocrystalline composed of a mixture of nanorods, nanoplates and nanoparticles	acetaldehyde	wet chemical	180	20–100	-	[35]
CuO nanoparticles	H_2_S	hydrothermal	40	0.2–5	297.5/54 (5 ppm)	[33]
ZnO nanofibers	formaldehyde	electrospinning	RT (under 365 nm UV-light)	5–100	32/17 (100 ppm)	[25]
WO_3_ nanoflowers	acetylene	hydrothermal	275	1–20	12/17 (200 ppm)	[27]
ZnO nanofibers	CO	electrospinning	700	1–5	-	[20]
NiO nanosphere	acetone	solvothermal	290	5–100	25/232 (200 ppm)	[56]
**Modification by noble and other metal particles**						
Pt/ZnO dense films,	H_2_	magnetron sputtering	RT	250–1000	23/43 (1000 ppm)	[15]
Pt/ZnO nanoparticles	H_2_	sol–gel	RT	250–1000	36/113 (1000 ppm)	[15]
Pt/ZnO nanorods	H_2_	hydrothermal	RT	250–1000	47/48 (1000 ppm)	[15]
Ag/ZnO nanorods	NO_2_	hydrothermal	225	1–50	<120/<150 (for every test concentration of NO2)	[58]
Au nanoparticles/SnO_2_ film	NO_2_	sputtering followed by annealing	RT	0.6–11	70/- (10 ppm)	[13]
Au@ZnO nanorods	acetone	microwave-assisted hydrothermal	150	0.005–100	8/5 (100 ppm)	[74]
Pd@ ZnO nanorods	acetone	microwave-assisted hydrothermal	150	0.005–100	9/7 (100 ppm)	[74]
Pt/ZnO microspheres composed of nanosheets	triethylamine	hydrothermal	200	8–100	15/70 (100 ppm)	[75]
**Hybrid structures**						
Application of several metal oxide						
NiO-ZnO hybrid microspheres	acetone	solvothermal	260	5–100	8/13 (200 ppm)	[56]
NiO/ZnO hexagonal nanostructure	acetone	controlled precipitation	330	5–100	-	[87]
CuO-ZnO	H_2_S	liquid phase	175	0.8–10	941/- (10 ppm)	[88]
SnO_2_-TiO_2_ yolk double-shelled microspheres	ethanol	Stöber method combined with hydrothermal	300	10–200	1.7/13.6 (200 ppm)	[57]
Ag-modified In_2_O_3_/ZnO nanobundles	formaldehyde	hydrothermal	300 100	0.1–0.8 0.1–1.6	6/3.6 (0.1 ppm) 12/6 (0.1 ppm)	[85]
Pd-doped CoTiO_3_/TiO_2_ nanoflowers	benzene	hydrothermal and calcination	RT	0.1–50	49/9 (0.5 ppm)	[91]
In_2_O_3_/NiO	NO_2_	atomic layer deposition (ALD)	145	0.5–50	14.3/6.54 (10 ppm)	[92]
Application of carbon-based nanomaterials						
Sn-TiO_2_@rGO/CNT nanocomposites	NH_3_	solvothermal	RT	25–250	99/66 (250 ppm)	[110]
SnO_2_-CuO nanoparticles/rGO nanosheets	NO_2_	hydrothermal	RT	5–50	90/255 (50 ppm)	[111]
NCD-ZnO-pSi hybrid structure	CO_2_	precipitation/drop casting	100 and 200	5–15	19/- (-)	[124]
SnO_2_QNP/ZnO nanosheets	H_2_S	sputtering and hydrothermal	RT	0.025–5	14/13 (0.1 ppm)	[125]
SnO_2_ QDs@MoS_2_ nanoflowers	NH_3_	solvothermal	RT	25–500	6/121 (100 ppm)	[126]
Modification with two-dimensional materials						
WS_2_ nanosheets	NH_3_	screen printing and microdrop	147	50–500	200/271.9 (250 ppm)	[137]
MoS_2_/Co_3_O_4_ nanocomposite	NH_3_	layer-by-layer self-assembly	RT	0.1–5)	98/100 (5 ppm)	[135]
CuO nanorods/MoS_2_ nanosheets	H_2_S	layer-by-layer self-assembly	RT	0.001–100	26/18 (30 ppm)	[136]
SnS_2_/SnO_2_ nanoflowers	NO_2_	microwave followed by in-situ thermal oxidation	100	0.125–4	299/143 (1 ppm)	[138]
MoO_3_ nanorods/MoSe_2_ nanoflowers	trimethylamine	hydrothermal and spin-coating	RT	0.02–1	12/19 (1 ppm)	[139]
Application of conducting polymers						
PANI/TiO_2_ nanocomposite	NH_3_ CO	polymerization	RT	23–114 23–114	10/30 (70 ppm)	[152]
PANI@SnO_2_ nanoparticles	NH_3_	polymerization	RT	10–100	33/- (100 ppm)	[149]
PANI-WO_3_ nanocomposite	NH_3_	polymerization	RT	1–100	30/170 (100 ppm)	[150]
PANI/SnO_2_ nanocomposite	H_2_	polymerization	RT	6000	11/7 (6000 ppm)	[147]
PANI/SnO_2_ nanocomposite	NH_3_	polymerization	RT	10–30	-/140 (10 ppm)	[156]
PANI/SnO_2_/rGO nanocomposite	NH_3_	polymerization	RT	10–30	2–5/80 (10 ppm)	[156]

## Data Availability

Not applicable.
